# Association Between Vitamin D Supplementation and Urolithiasis Recurrence Outcomes in Known Stone Formers: A Retrospective Cohort Study With Dose-Response Analysis

**DOI:** 10.7759/cureus.90853

**Published:** 2025-08-24

**Authors:** Nawaf Alzaben, Abdulrahman Mokhtar, Faisal Altwijri, Saleh Alawaji, Raed Almannie, Saleh Binsaleh, Abdulaziz Althunayan

**Affiliations:** 1 Department of Urology, King Saud University, Riyadh, SAU; 2 Department of Urology, King Khalid University Hospital, Riyadh, SAU; 3 Department of Radiology, King Saud University, Riyadh, SAU

**Keywords:** dose-response relationship, retrospective cohort, stone recurrence, urolithiasis, vitamin d

## Abstract

Background

Vitamin D supplementation is widely prescribed to correct vitamin D deficiency, but its impact on urinary stone recurrence in patients with a history of urolithiasis (stone formers) remains uncertain.

Methods

We conducted this retrospective cohort study at King Khalid University Hospital, Riyadh, Saudi Arabia. A total of 353 adult stone formers were categorized as non-supplemented (n = 235) or supplemented (n = 118), with stratification by dose (≤800 IU daily, 10,000 IU weekly, and 50,000 IU weekly). The primary outcome was the recurrence of urolithiasis. Secondary outcomes included time-to-recurrence and dose-response. Statistical methods included chi-square testing, Kaplan-Meier analysis, subgroup comparison, and multivariable logistic regression. Clinical and laboratory data were extracted from electronic medical records using a standardized data collection form.

Results

The mean age of patients was 45.5 ± 14.3 years, with males comprising 68.3% of the cohort. Recurrence occurred in 32.8% of non-supplemented and 45.8% of supplemented patients (absolute risk increase = 13.0%). Multivariable analysis showed higher odds of recurrence with supplementation (adjusted OR = 1.65, 95% CI: 1.01-2.71; p = 0.047). Recurrence rates differed significantly by dose category (p = 0.049), with the highest in the 50,000 IU group. Kaplan-Meier analysis showed no significant difference in time-to-recurrence (p = 0.071).

Conclusions

In this retrospective cohort of known stone formers, vitamin D supplementation, particularly at higher doses, was associated with a greater risk of recurrence. These findings support the possible need for a more individualized, dose-conscious approach to supplementation in stone-prone patients.

## Introduction

Urolithiasis, or urinary stone disease, is a globally prevalent condition with high recurrence rates and a significant clinical and economic burden. Lifetime prevalence in the United States is estimated to be 10.1%, while studies in Saudi Arabia report even higher rates, ranging from 13% to 19% [1,2]. Known risk factors include low fluid intake, genetic predisposition, and dietary patterns [3].

Among individuals who experience an initial stone episode, recurrence affects approximately 50% within 5-10 years [4]. Consequently, secondary prevention strategies are a critical component of long-term management. Vitamin D is essential for calcium and phosphate homeostasis and has emerged as a point of clinical interest due to its widespread use and complex interaction with renal physiology. Hypovitaminosis D is prevalent across the Middle East despite plentiful sunshine, often necessitating supplementation [5,6].

Vitamin D enhances intestinal calcium absorption, which may increase urinary calcium excretion, a known lithogenic factor. This mechanism raises concerns about stone risk, particularly in individuals predisposed to hypercalciuria. While observational studies in healthy populations suggest that typical vitamin D intake does not significantly increase nephrolithiasis risk [7], these findings may not be generalizable to patients with a confirmed history of urolithiasis (i.e., stone formers), who may have underlying metabolic derangements. Evidence overall remains inconclusive: some studies have identified an independent association between elevated plasma 1,25-dihydroxyvitamin D levels, even within physiologic ranges, and an increased risk of symptomatic kidney stone formation [8], whereas others report neutral or inconsistent outcomes, often attributing discrepancies to differences in study design, dosing regimens, baseline risk, and follow-up duration [9,10]. Meta-analyses have likewise failed to provide definitive conclusions, and current clinical guidelines do not offer consistent recommendations regarding vitamin D supplementation in patients with a history of urolithiasis [11].

Despite the biological plausibility and increasing clinical relevance, there remains a paucity of targeted research evaluating the relationship between vitamin D supplementation and stone recurrence in individuals with a history of urolithiasis. Dose-response data are scarce, and the impact of supplementation on recurrence risk remains unclear in this vulnerable subgroup.

To address this gap, we conducted a retrospective cohort study of adult patients with a confirmed history of urolithiasis with the following objectives: (1) to determine the association between vitamin D supplementation and the risk of stone recurrence (primary objective); (2) to compare time to recurrence between supplemented and non-supplemented patients (secondary objective); and (3) to explore dose-response relationships by stratifying outcomes according to supplementation dose (secondary objective).

## Materials and methods

Study design and population

**Figure 1:**
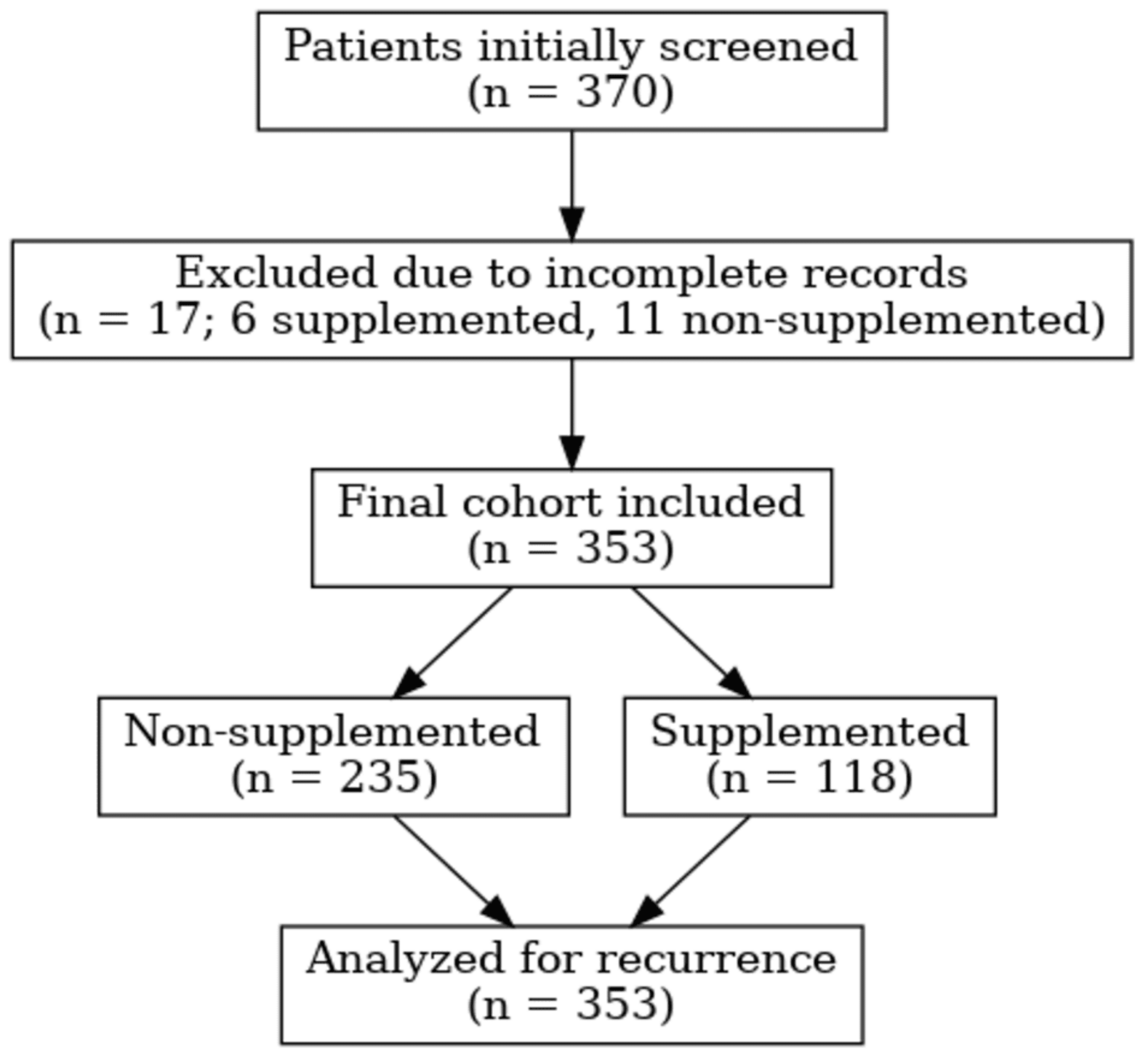
Cohort flow diagram

We conducted a single-center, retrospective cohort study involving adults (≥18 years) with documented urolithiasis from 2020 to 2025. After excluding incomplete records, the final dataset included 353 patients (Figure 1).

Institutional review board approval was waived for the analysis of de-identified data. Vitamin D supplementation status and dosage (in IU) were extracted from patients' electronic medical records. Patients were categorized into two groups: non-supplemented and supplemented, with the supplemented group further stratified into doses of ≤800 IU daily, 10,000 IU weekly, or 50,000 IU weekly.

Outcomes

The primary outcome was stone recurrence, defined as evidence of a new stone six or more months after the index episode. Secondary outcomes were time-to-recurrence and dose-response within the supplemented cohort. A supplemented patient was defined as one who had received at least 30 days of vitamin D supplementation, which at our institution was routinely prescribed in the form of cholecalciferol.

Covariates

Demographic and clinical covariates included age, sex, BMI, and estimated glomerular filtration rate (eGFR). Follow-up extended to last clinic visit or end of study date (median = 24 months).

Sample size calculation

A priori sample size calculation was conducted to estimate the number of subjects required to detect a clinically meaningful difference in stone recurrence between vitamin D-supplemented and non-supplemented patients. Based on prior literature, the estimated recurrence rate in the non-supplemented group was assumed to be approximately 25%. In comparison, the supplemented group was expected to have a recurrence rate of around 35-40%, representing a 10-15% absolute increase.

To detect this difference in proportions with 80% power and a two-sided alpha of 0.05, assuming a 2:1 ratio of non-supplemented to supplemented patients, the required total sample size ranged from 216 to 384 subjects, depending on the assumed effect size.

For the time-to-event analysis (Kaplan-Meier survival comparison), sample size was also estimated using the log-rank test. Assuming a hazard ratio between 1.5 and 1.7 for recurrence in the supplemented group relative to the non-supplemented group, with 80% power and an overall recurrence event rate of 30% over the follow-up period, the required sample size ranged from 307 to 578 total subjects.

The final cohort included 235 non-supplemented and 118 supplemented patients (total n = 353), meeting the required sample size for detecting a 10-15% difference in recurrence rate and allowing for adequately powered time-to-event analysis.

Statistical analysis

Continuous variables were summarized using means and standard deviations and compared using independent samples t-tests. Categorical variables were expressed as counts and percentages and analyzed using chi-square or Fisher’s exact tests where appropriate.

Multivariable logistic regression was employed to assess the association between vitamin D supplementation and recurrence of urolithiasis, adjusting for age, sex, BMI, and eGFR. Adjusted odds ratios (ORs) and 95% confidence intervals (CIs) were reported. To explore a potential dose-response relationship, recurrence rates were compared across three supplementation categories (≤800 IU, 10,000 IU, and 50,000 IU) using chi-square testing.

Time-to-recurrence was evaluated with Kaplan-Meier survival analysis, and differences between groups were tested using the log-rank test. A two-sided p-value of <0.05 was considered statistically significant. All statistical analyses were conducted using Python (version 3.11; Python Software Foundation, Wilmington, DE).

## Results

Cohort characteristics

Of 353 patients, 235 (66.6%) were non-supplemented, and 118 (33.4%) received vitamin D. Baseline age, BMI, eGFR, and serum calcium were comparable between groups; the non-supplemented group had a higher proportion of males (Table 1).

**Table 1:** Baseline characteristics by supplementation status n: Number, SD: Standard Deviation, BMI: Body Mass Index, eGFR: Estimated Glomerular Filtration Rate

Variable	Overall (n = 353)	Non-supplemented (n = 235)	Supplemented (n = 118)
Age (mean ± SD)	45.5 ± 14.3	44.5 ± 14.3	47.6 ± 14.1
Male (%)	68.3%	73.6%	57.6%
BMI (mean ± SD)	29.1 ± 5.8	29.0 ± 5.8	29.2 ± 5.8
eGFR (mean ± SD)	91.5 ± 26.4	89.9 ± 27.2	94.7 ± 24.6
Serum Calcium (mmol/L)	2.31	2.31	2.31

The median follow-up duration was 29.8 months in the supplemented group and 22.6 months in the non-supplemented group.

Recurrence rates

Among the 353 patients, recurrence occurred in 131 cases (37.1%). Recurrence occurred in 32.8% of non-supplemented and 45.8% of supplemented patients, yielding an absolute risk increase of 13.0%. This difference was statistically significant (chi-square p = 0.023) (Table 2).

**Table 2:** Recurrence outcomes by supplementation status n: Number

Variable	Non-supplemented (n = 235)	Supplemented (n = 118)	Overall (n = 353)
No Recurrence	158	64	222
Recurrence	77	54	131
Recurrence Rate (%)	32.8%	45.8%	37.1%

**Figure 2:**
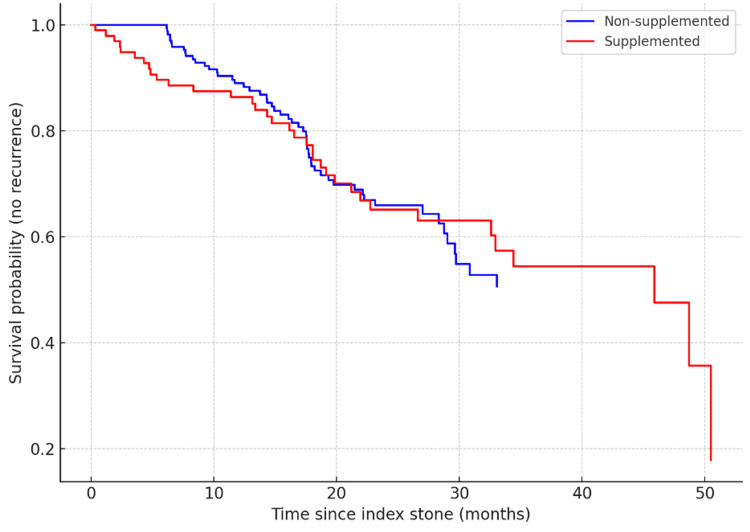
Kaplan-Meier survival curves comparing time to stone recurrence between supplemented and non-supplemented patients

Kaplan-Meier analysis showed no significant difference in time-to-recurrence between groups (log-rank = 1.81, p = 0.071) (Figure 2, Table 3).

**Table 3:** Time to recurrence by supplementation status

Group	Median Time to Recurrence (Months)	Log-Rank p-value
Non-supplemented	15.0	0.071
Supplemented	15.6	0.071

Dose response

**Figure 3:**
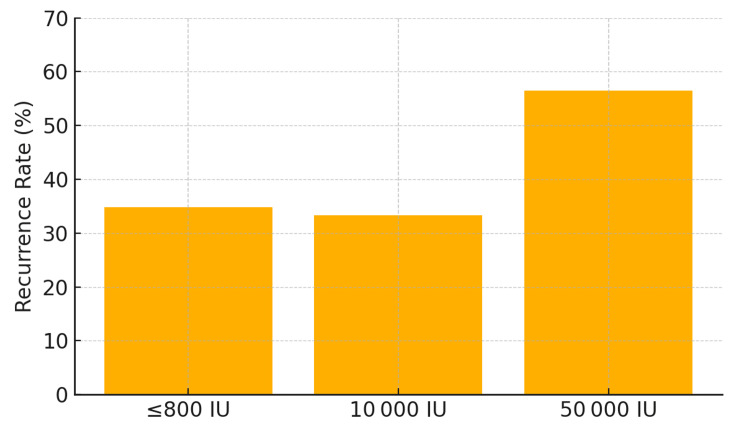
Bar plot showing recurrence rates among supplemented patients stratified by vitamin D dose IU: International Units

Within supplemented patients, recurrence rates were 34.8% for ≤800 IU, 33.3% for 10,000 IU, and 56.5% for 50,000 IU (Figure 3). The chi-square test across dose categories was significant (p = 0.049) (Table 4).

**Table 4:** Recurrence by dose category in supplemented patients IU: International Units

Dose Category	No Recurrence	Recurrence	Total	Recurrence Rate (%)
≤800 IU	15	8	23	34.8%
10,000 IU	22	11	33	33.3%
50,000 IU	27	35	62	56.5%
All Combined	64	54	118	45.8%

Multivariable logistic regression

**Figure 4:**
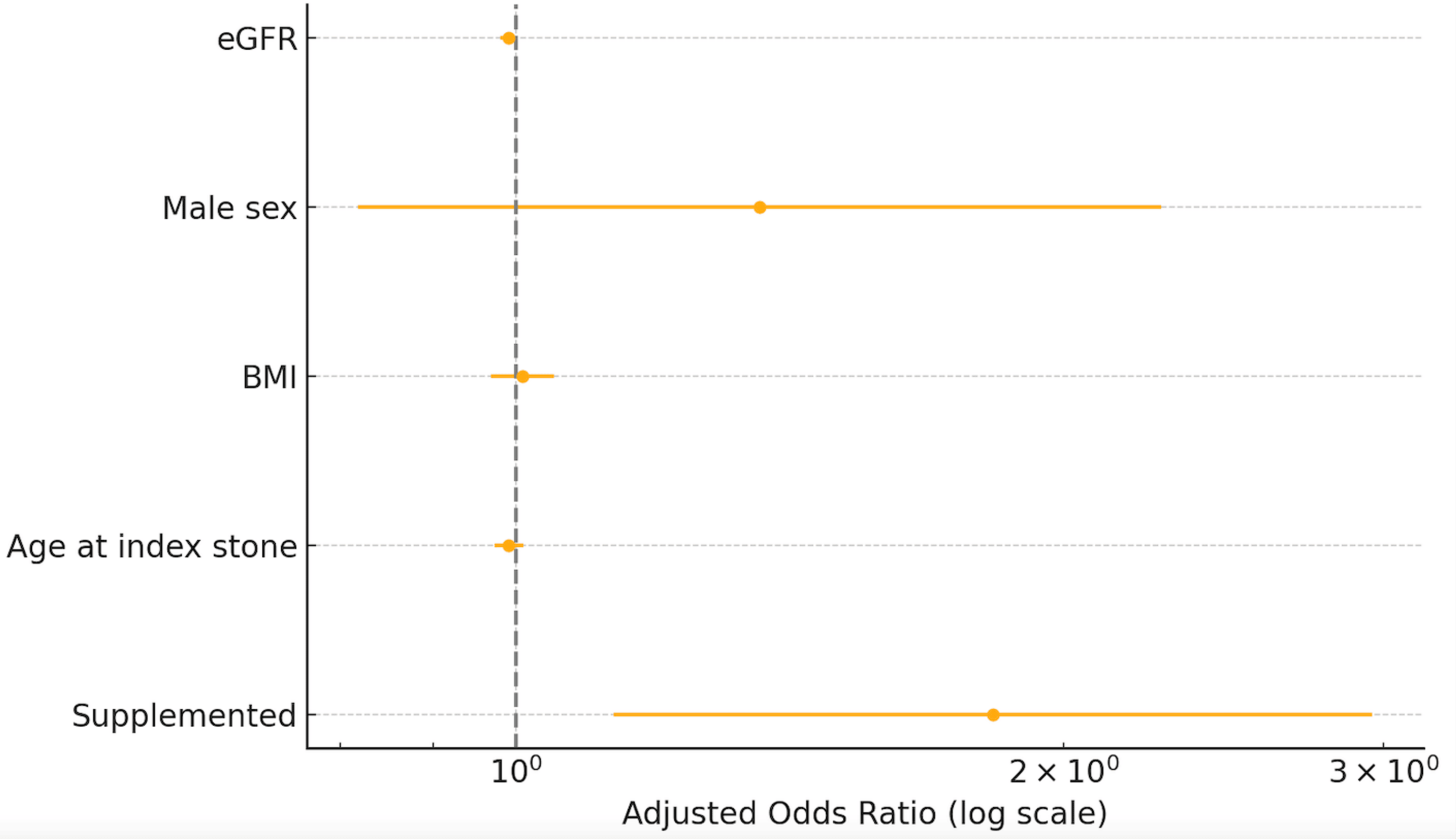
Forest plot showing adjusted odds ratios (OR) for stone recurrence from the multivariable logistic regression model eGFR: Estimated Glomerular Filtration Rate, BMI: Body Mass Index

After adjustment for confounders, vitamin D supplementation remained associated with higher recurrence odds (adjusted OR = 1.83, 95 % CI: 1.13-2.96; p = 0.014). Age, BMI, eGFR, and male sex were not independently associated (Figure 4, Table 5).

**Table 5:** Adjusted odds of recurrence (multivariable logistic regression) OR: Odds Ratio, CI: Confidence Interval, BMI: Body Mass Index, eGFR: Estimated Glomerular Filtration Rate

Variable	Adjusted OR	95% CI	p-value
Supplemented	1.83	1.13-2.96	0.014
Age	0.99	0.97-1.01	0.337
BMI	1.01	0.97-1.05	0.706
eGFR	0.99	0.98-1.00	0.052
Male Sex	1.36	0.82-2.26	0.235

## Discussion

In this retrospective analysis of 353 individuals with a history of urolithiasis, we observed a statistically significant, dose-dependent association between vitamin D supplementation and stone recurrence. Our primary finding was that supplemented patients had higher odds of a subsequent stone event (adjusted OR = 1.83, 95% CI: 1.13-2.96), suggesting that routine vitamin D repletion in this high-risk population may warrant closer scrutiny and a more individualized approach. The observed dose-response pattern, with recurrence rates the highest in the 50,000 IU group (56.5%), lends additional support to the biological plausibility of this association.

Our findings align with a growing body of evidence suggesting that the effects of vitamin D on urinary calcium excretion may not be benign, particularly in patients with a history of urolithiasis who may have underlying metabolic predispositions. While large-scale studies in the general population have not demonstrated a clear link between modest vitamin D intake and incident stones [12], our study focuses on a more vulnerable group: recurrent stone formers. This distinction is critical. Patients with a history of urolithiasis often exhibit baseline abnormalities such as idiopathic hypercalciuria, which can be exacerbated by vitamin D-mediated increases in intestinal calcium absorption [13,14]. A 2021 systematic review and meta-analysis concluded that vitamin D supplementation significantly increases the risk of hypercalciuria and hypercalcemia, both established risk factors for calcium-based stones [12]. This aligns with our observation that high-dose supplementation may confer the most significant risk.

The dose-dependent nature of our results is a key finding and resonates with recent clinical investigations. For instance, a 2021 prospective study found that correcting vitamin D deficiency with high-dose cholecalciferol led to a significant rise in 24-hour urinary calcium levels, prompting the authors to recommend caution [15]. Our data, showing a sharp increase in recurrence with 50,000 IU doses compared to lower doses (≤800 IU or 10,000 IU), provides real-world evidence that the magnitude of vitamin D exposure may be a critical determinant of outcome in secondary stone prevention.

What distinguishes our study is its focus on clinically relevant recurrence outcomes within a cohort of known stone formers stratified by real-world dosing regimens. While previous studies have focused primarily on biochemical endpoints such as hypercalciuria [14,15] or incident stones in healthy cohorts [12], our work directly links supplementation to the tangible outcome of stone recurrence. The trend towards earlier recurrence in the supplemented group, although not reaching statistical significance in the Kaplan-Meier analysis (log-rank p = 0.071), complements the significant findings from our adjusted regression model and dose-stratified analysis. This suggests that, while the overall time-to-event difference may be subtle, the cumulative risk over time is substantially increased by supplementation, particularly at high doses.

Although males comprised 68.3% of our study population, gender was not found to be a statistically significant variable in the multivariable logistic regression (p = 0.235). This aligns with the findings of a 2024 study, which reported that in multivariate analysis, sex did not show significant differences in the recurrence of calcium oxalate stones (p = 0.099) [16].

These findings carry particular relevance for Saudi Arabia, given the high prevalence of vitamin D deficiency in the population and the frequent use of supplementation as a corrective measure. However, this same local context makes our results less generalizable. The association we found between supplementation and urolithiasis recurrence might not apply to populations in other areas where vitamin D deficiency is less common.

This study has several notable strengths. First, it examines patients with a confirmed history of urolithiasis, a high-risk group often underrepresented in prior research on vitamin D and stone risk. By focusing on this clinically relevant population, the findings provide context that may be more directly informative for stone formers than studies conducted in general populations. Second, the cohort was stratified not only by supplementation status but also by dose, allowing both group-wise comparisons and exploratory dose-response assessment, an aspect that has been rarely examined in this field. Third, recurrence outcomes were defined using standardized clinical criteria and verified through electronic medical records, supporting reliable outcome ascertainment. Fourth, all patients had documented follow-up within a tertiary care setting, reducing the likelihood of missing recurrence events and strengthening internal validity. Finally, the study contributes data from a Middle Eastern population, where vitamin D deficiency is highly prevalent, thereby adding insight from a setting with distinct baseline risk characteristics.

Limitations of this study include its retrospective design, single-center scope within a relatively homogenous Arab population, the absence of 24-hour urine data, and the lack of baseline or follow-up 25(OH)D levels. The supplemented group also had a longer median follow-up duration (29.8 vs. 22.6 months), which may have increased recurrence detection over time. While this reflects real-world variability in follow-up, it introduces potential surveillance bias that could overestimate the association between supplementation and recurrence risk. Another limitation is the discrepancy between analytic methods: logistic regression demonstrated a significant association after covariate adjustment, whereas Kaplan-Meier, which accounts for censoring and event timing but is unadjusted, did not reach significance. This inconsistency highlights the need for cautious interpretation, as different statistical approaches yield different insights.

Furthermore, our definition of supplementation (≥30 days) did not account for cumulative exposure or long-term adherence, and available records did not permit categorization by duration. This may have diluted dose-response effects, as short-term users differ from long-term users. Similarly, we did not perform a time-to-recurrence comparison across all four dose groups, as subgroup sizes were too small to allow sufficient statistical power. As with many retrospective studies, adherence to supplementation could not be directly verified, and baseline or follow-up 25(OH)D levels were not consistently documented. Together, these limitations may have influenced recurrence risk estimates and underscore the need for larger, prospective studies with systematic measurement of dose, duration, adherence, and biochemical parameters, ideally incorporating Cox proportional-hazards models to better evaluate time-to-event outcomes.

Clinical implications

These findings may have implications for the management of stone-forming patients. Vitamin D repletion remains appropriate in cases of documented deficiency, but caution may be warranted with high-dose regimens in individuals with a history of urolithiasis. Where higher doses are clinically necessary, closer follow-up and urinary monitoring could help reduce potential risk. Lower daily doses might represent a safer option, though individualized assessment remains essential.

## Conclusions

Vitamin D supplementation, particularly at higher doses, was associated with a higher observed recurrence rate in patients with a history of urolithiasis. These findings highlight the possible need for more tailored supplementation strategies and support the call for prospective trials to better define safe dosing thresholds in stone-prone populations.

## References

[1] Chen Z, Prosperi M, Bird VY (2018). Prevalence of kidney stones in the USA: the National Health and Nutrition Evaluation Survey. J Clin Urol.

[2] Alhubaishy BA, Bokhary OA, Alhuzali MA, Bokhary HA (2024). Prevalence of urolithiasis in Saudi Arabia: a systematic literature review. Urol Ann.

[3] Sorokin I, Mamoulakis C, Miyazawa K, Rodgers A, Talati J, Lotan Y (2017). Epidemiology of stone disease across the world. World J Urol.

[4] Rule AD, Lieske JC, Li X, Melton LJ 3rd, Krambeck AE, Bergstralh EJ (2014). The ROKS nomogram for predicting a second symptomatic stone episode. J Am Soc Nephrol.

[5] Palacios C, Gonzalez L (2014). Is vitamin D deficiency a major global public health problem?. J Steroid Biochem Mol Biol.

[6] Bassil D, Rahme M, Hoteit M, Fuleihan G (2013). Hypovitaminosis D in the Middle East and North Africa: prevalence, risk factors and impact on outcomes. Dermatoendocrinol.

[7] Ferraro PM, Taylor EN, Gambaro G, Curhan GC (2017). Vitamin D intake and the risk of incident kidney stones. J Urol.

[8] Taylor EN, Hoofnagle AN, Curhan GC (2015). Calcium and phosphorus regulatory hormones and risk of incident symptomatic kidney stones. Clin J Am Soc Nephrol.

[9] Zhang F, Li W (2024). The complex relationship between vitamin D and kidney stones: balance, risks, and prevention strategies. Front Nutr.

[10] Schulster ML, Goldfarb DS (2020). Vitamin D and kidney stones. Urology.

[11] Cosman F, de Beur SJ, LeBoff MS, Lewiecki EM, Tanner B, Randall S, Lindsay R (2014). Clinician’s guide to prevention and treatment of osteoporosis. Osteoporos Int.

[12] Malihi Z, Wu Z, Stewart AW, Lawes CM, Scragg R (2016). Hypercalcemia, hypercalciuria, and kidney stones in long-term studies of vitamin D supplementation: a systematic review and meta-analysis. Am J Clin Nutr.

[13] Letavernier E, Daudon M (2018). Vitamin D, hypercalciuria and kidney stones. Nutrients.

[14] Charoenngam N, Holick MF (2020). Immunologic effects of vitamin D on human health and disease. Nutrients.

[15] Taheri M, Tavasoli S, Shokrzadeh F, Amiri FB, Basiri A (2019). Effect of vitamin D supplementation on 24-hour urine calcium in patients with calcium urolithiasis and vitamin D deficiency. Int Braz J Urol.

[16] Cheng WY, Tseng JS (2024). Urinary stone analysis and clinical characteristics of 496 patients in Taiwan. Sci Rep.

